# Formamint in the Treatment of Oral Conditions

**Published:** 1912-10-05

**Authors:** 


### FORMAMINT IN THE TREATMENT OF ORAL CONDITIONS.

By A LATE SHIP'S SURGEON.

One of the most frequent conditions that the ship's surgeon is called upon to treat is syphilis, either in its primary or subsequent manifestations. Although the treatment is generally easy, especially now that we have excellent adjuncts in the newer synthetic remedies, there are some points which are in danger of being overlooked. One of these is the importance of securing oral asepsis. Every one of us knows that the condition of the teeth and of the mouth demands attention in a patient who is subjected to drastic mercurial treatment. On board ship it is not always easy to secure such scrupulous cleanliness, especially where members of the crew are concerned. A tooth-brush and a simple mouth-wash, such as is prescribed at Aix, are generally sufficient if the patient uses both as frequently and as carefully as he is directed. But it is not always the case that the surgeon's directions are carried out, and once the teeth, gums, and mucous membrane of the mouth are infected, considerable difficulty may be experienced in remedying the condition. In such cases I have found formamint of great value. The investigations of Piorkowski and others have shown that this preparation possesses marked bactericidal properties, especially so far as the *Bacillus influenzæ* is concerned. It is, as is generally known, an agreeable way of supplying an oral germicide, but it is not usually to be found in the ship's medicine chest.

In syphilitic cases my practice has been to supply the patient with a couple of dozen tablets and to direct him to suck one or two frequently during the day. A simple and efficient mouthwash is made by dissolving a dozen tablets in ten ounces of water, adding a few drops of tincture of myrrh and a couple of drams of glycerine. If the mouth is rinsed out thrice daily with this solution, and a soft tooth-brush is used at the same time, no difficulty will be found in securing asepsis. The simpler and easier method, however, is to give the tablets, which may be sucked while the sailor is on duty, and which appear to be highly efficient.

The preparation serves equally well in those cases of septic sore throat which are frequently seen in debilitated patients, especially in those who have suffered severely from sea-sickness. Ordinarily these cases are annoyances; they resist treatment for many days and clear up only when the patient has regained strength. The cure may be much hastened by prescribing formamint, and by painting the tonsils lightly with a weak solution of iodine. As a tonic the triple phosphate syrup in dram doses three times a day is an excellent adjuvant. Cultures made from the tonsils in such cases show usually a rich growth of streptococci, and it is an interesting fact that these clear up very rapidly under formamint treatment.

I have had equally good results in the treatment of oral and faucal ulceration in malaria patients. It is usually stated that such ulceration is "malarial"; in the East, for instance, a malarial sore throat is frequently diagnosed, perhaps generally on quite insufficient grounds. The organism found in the excavated ulcers on the floor of the mouth or on the tonsils is nearly always a streptococcus, and it is probable that these lesions are ordinary streptococcal sore throat, or ulceration supervening in patients whose vitality has been much lowered by the attacks of malaria. The ulceration on the floor of the mouth, generally encroaching on the frenum linguæ, is sometimes very intractable; in old patients, the thick, rounded edges and slight marginal induration may give rise to a suspicion of malignancy.

After taking the usual precautions, such as removing any irritating jagged tooth or rasping tooth-plate, and attending to the patient's diet, treating any gastric disturbance that may be present, the treatment resolves itself into sterilising the mouth and promoting healing of the ulcer by soothing applications. After cocainising the site, it is advisable, in the majority of cases, to apply a solution of silver nitrate (ten grains to the ounce) or of tincture of iodine (eight per cent) to the ulcer itself, but even then the ulcers take a long time to heal, and there is frequently considerable scarring. It is in such cases that I have found formamint of decided value. By frequently sucking a pellet, and making the formaldehyde saliva thoroughly flush the floor of the mouth, so that it comes into direct contact with the ulcer, a constant, mild, germicidal action is secured which decidedly promotes healing. The patient should continue the use of the tablets for some time after the ulcers have healed, as a recurrence is by no means infrequent, especially in debilitated patients.

There are many other oral conditions in which, on board ship, the use of formamint is advisable, and as there are few drugs more easy to prescribe or more readily taken by patients, the provision of these tablets is almost a necessity for the ship's surgeon. In the tropics especially they will be found valuable. Their frequent use in cases of "sailor's sprue," in which sometimes the condition of the mouth almost baffles description, tends to ease the patient and to make him more comfortable, even though they cannot be said to be curative. As a prophylactic against influenza, diphtheria, and sore throat generally they are too well known to require commendation, but it has seemed to me that their use in the oral conditions above indicated is not so generally recognised.

